# The Role of Dupilumab in Severe Asthma

**DOI:** 10.3390/biomedicines9091096

**Published:** 2021-08-27

**Authors:** Fabio Luigi Massimo Ricciardolo, Francesca Bertolini, Vitina Carriero

**Affiliations:** Department of Clinical and Biological Sciences, University of Turin, San Luigi Gonzaga University Hospital, Orbassano, 10043 Turin, Italy; francesca.bertolini@unito.it (F.B.); vitina.carriero@unito.it (V.C.)

**Keywords:** severe asthma, IL-4, IL-13, dupilumab, phenotype

## Abstract

Dupilumab is a fully humanized monoclonal antibody, capable of inhibiting intracellular signaling of both interleukin (IL)-4 and IL-13. These are two molecules that, together with other proinflammatory cytokines such as IL-5 and eotaxins, play a pivotal role in orchestrating the airway inflammatory response defined as Type 2 (T2) inflammation, driven by Th2 or Type 2 innate lymphoid cells, which is the major feature of the T2 high asthma phenotype. The dual inhibition of IL-4 and IL-13 activities is due to the blockade of type II IL-4 receptor through the binding of dupilumab with the subunit IL-4Rα. This results in the repression of STAT6 and in the suppression of subsequent de novo formation of several molecules involved in the T2 inflammatory signature. Several clinical trials tested the efficacy and safety of dupilumab in large populations of uncontrolled severe asthmatics, revealing significant improvements in lung function, asthma control, and exacerbation rate. Similar results were reported when dupilumab was employed in patients harboring pathogenetic processes related to T2 immune response, such as atopic dermatitis and chronic rhinosinusitis. In this review, we provide an overview of the recent research in the field of respiratory medicine about dupilumab mechanism of action and its effects.

## 1. Introduction

Asthma is a complex and heterogeneous condition defined by symptoms (wheeze, shortness of breath, chest tightness, and cough) and airflow obstruction that vary over time [1,2]. Asthma is associated with chronic inflammatory status, airway hyperresponsiveness, and structural changes of the airways that are called airway remodeling [3]. Asthma represents the most common chronic disease, affecting 300 million individuals globally (Global Initiative for Asthma 2017) and was responsible for 495,000 deaths worldwide in 2017 [4]. Traditionally, asthma was simply differentiated as either extrinsic (atopic) or intrinsic (non-atopic) [5]. Over the last 20 years, researchers have identified several phenotypes that are the observable results of the interactions between environmental and genetic factors and are defined by the association of clinical, biological, and pathophysiological characteristics. In the last decade, the classification of asthma heterogeneity has improved due to the development of more accurate tools for assessing disease characteristics that highlighted the discrepancy in clinical, physiological, and pathological markers. Moreover, the great complexity of asthma is reflected by the fact that each phenotype is underpinned by multiple endotypes that are defined as distinct molecular mechanisms determined by genetic factors [6].

The recent clustering studies, including the Leicester Study, the Severe Asthma Project (SARP), the Airway Disease Endotyping for Personalized Therapeutics (ADEPT), and the Unbiased Biomarkers for the Prediction of Respiratory Disease Outcome Consortium (UBIOPRED), were able to identify distinct phenotypic groups that exhibited clinically and relevant pathophysiological differences in such factors as lung function, atopy, sex, symptoms, age of asthma onset and duration, treatment use, inflammatory pattern, and health care utilization [2,7,8,9]. These studies led to the determination of the two major asthma phenotypes, defined based on the inflammatory status of the patients T2-mediated airway inflammation, known as T2 high asthma, and T2 low asthma. Despite the novel knowledge in phenotyping and endotyping asthma, asthma pathophysiology is not completely clarified. The first line of therapy is represented by inhaled corticosteroids (ICS), which are administered at different doses based on disease severity. Asthmatic patients who share similar clinical symptoms may show different responses to the same ICS treatment, this being due to the progressive heterogeneity of asthma, which is recognized as a key feature of this disease. In particular, a subset of asthmatic patients that fail to control their disease despite the higher ICS dose treatment or combination with other second controller medication (tiotropium, oral corticosteroids, leukotriene modifiers) are defined, by the ERS/ATS Guidelines, as having severe asthma [10,11]. Within the plethora of types of asthma and the different responses to the therapy, severe asthmatics are the group with the most pressing need of new therapy and can be eligible to add-on biological therapies [12]. These are therapies derived from the development of a new target of interventions on a specific pathway of the asthma endotypes that include immunoglobulins E (IgE) and interleukin-5 (IL-5) and its receptor, as well as the IL-4 receptor [12]. On this basis, the characterization of the complex mechanisms underlying the inflammatory phenotypes through specific, sensitive, and easy to obtain biomarkers plays a prominent role in establishing better-personalized therapy. This review aims to discuss the role of dupilumab, a human anti-interleukin-4 receptor α subunit monoclonal antibody that blocks both IL-4 and IL-13 signaling in severe asthma.

## 2. T2 High Mechanisms

Historically, type 2 (T2) asthma has been associated with a predominance of eosinophilic inflammation, atopy, and an immune response mediated by T helper 2 (Th2) lymphocytes, leading to the definition of Th2 asthma. Subsequent studies revealed that elevated eosinophilia in conjunction with type 2 response may not be associated with atopy.

The T2 high asthmatic phenotypes are associated with elevated expression of Th2 cytokines such as IL-5, IL-4, IL-9, and IL-13 and are classified as allergic and non-allergic. The former has generally an early-onset and is characterized by high levels of serum IgE targeted against specific antigens.

The triggering mechanism of allergic asthma takes place in the epithelium, which is composed of cells presenting several surface receptors, such as Toll-like receptors (TLRs), (NOD)-like receptors (NLRs), C-type lectin receptors (CLRs), retinoic acid-inducible gene (RIG)-I-like receptors (RLRs), protease-activated receptors, and purinergic receptors that are all activated by antimicrobial and/or environmental stimuli [13]. This induces the release of a plethora of chemokines and cytokines that recruit from the bone marrow to the airways a subset of cells called dendritic cells (DCs), capable of uptaking allergens and exposing on their surface peptides derived from allergens. The interaction between DCs presenting antigens and naïve T lymphocyte receptors leads to the differentiation in Th2 cells. Among the cytokines released by the damaged epithelium are included IL-33, IL-25, and thymic stromal lymphopoietin (TSLP), which are linked to T2 asthma, although their precise contribution is still unclear in humans.

**Figure 1:**
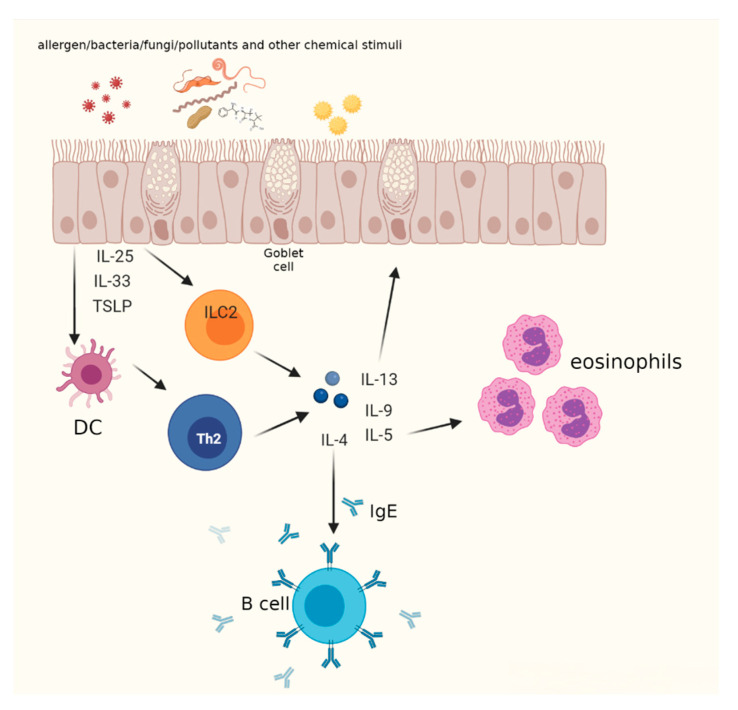
The role of Th2 and ILC2 in asthma. After injury, epithelial cells release allarmins (IL-25, IL-33, and TSLP) that activate ILC2 cells and dendritic cells (DC). Upon allergen/antigen uptake, processing, and presentation to naïve T cells, DC promote the differentiation of naïve T helper cells into Th2 lymphocytes. ILC2 and Th2 secrete pro-inflammatory cytokines, exerting key roles in T2 immune response. Created with BioRender.com (accessed on 23 July 2021).

Recently, the key role of type 2 innate lymphoid cells (ILC2) in the pathogenesis of asthma has been shown. These cells in response to the Th2 cell-stimulating cytokines IL-25, IL-33, and TSLP produced by epithelial cells release key Th2 cell-associated cytokines (IL-4, IL-5, IL-9, and IL-13) and other mediators of tissue growth, inflammation, and repair [14]. Based on this the term, Th2 asthma was changed to T2 asthma [15] (Figure 1).

Evidence states that IL-4, IL-5, and IL-13 drive allergic asthma, which explains the importance of these molecules as drug targets in the management of T2 high allergic asthma.

T2 high asthma is identified by high counts of airway eosinophil that can function as immunomodulator cells through the generation of a wide variety of cytokines, including IL-5. This cytokine mediates eosinophils recruitment and accumulation into the airways and their maturation, survival, and activation [16]. Much of the eosinophil’s involvement in airway dysfunction in asthma comes from the actions of IL-5, which has become an effective target in therapy. The first author who wrote about eosinophils was Paul Ehrlich, defining them as leucocytes with a bilobed nucleus [16]. Evidence obtained from animal models suggested the existence of two subsets of eosinophils in the lung, defined as resident and inflammatory eosinophils (rEosinophils and iEosinophils, respectively), that display differential expressions of surface biomarkers (Siglec F, CD11, Gr1, CD62L, CD125, CD101), nuclear morphology (ring-shaped in the rEosinophils and segmented in the iEosinophils), and intracellular content. Additional studies described a subset of eosinophils, called homeostatic (hEosinophils), that were different from inflammatory eosinophils because of their IL-5-independent differentiation and immunoregulatory action in reducing Th2 response [16].

In humans, cellular density analysis allowed two different groups of eosinophils to be distinguished, which were defined as normodense (in healthy lungs) and hypodense (in asthmatic lungs). The latter subgroup was also characterized by a minor content in granules and a greater response to activating stimuli in terms of survival, adhesion, oxygen metabolism, superoxide production, and antibody-dependent cytotoxicity. It is still debated whether the eosinophils in the lung can be considered as a distinct population from the circulating ones, although available evidence revealed differences between lung eosinophils and circulating cells, with the former displaying lower density, responsiveness, and surface marker expression [17].

Two interventional strategies are available to regulate IL-5 and the eosinophil’s participation in asthma. One is mepolizumab, a humanized monoclonal antibody directed against the human cytokine IL-5, and the other is benralizumab, a humanized monoclonal antibody that targets its receptor and acts by a dual mechanism [12,18].

The differentiation factor for driving T2 response is represented by IL-4. This cytokine initiates T cell differentiation towards the Th2 subtype and induces the production of T2-associated cytokines and chemokines, including IL-5, IL-9, IL-13, and eotaxins. IL-4 is capable of driving the class switching of B cell immunoglobulin towards IgE and IgG4 [19,20]. Moreover, this interleukin can induce the overexpression of the vascular cell adhesion molecule 1 (VCAM-1), which is involved in eosinophil recruitment from the bloodstream to the lungs through the interaction with α4-integrin [21].

IL-13 plays a major role in tissue remodeling, leading to mucus hypersecretion by goblet cells, fibrosis, smooth muscle alterations, and increased airway hyperactivity. In addition, IL-13, along with IL-4, induces the production of eosinophil-promoting factors, including IL-5 and eotaxin-3 from Th2 cells and epithelial cells [22,23]. Furthermore, IL-13 also acts as a potent inductor of the inducible isoform of the enzyme nitric oxide synthase (iNOS) in the airway epithelium. The amount of NO released by the bronchial epithelium is defined as fractional exhaled nitric oxide (F_E_NO), which is considered a T2 biomarker [24].

## 3. Mechanism of Action of Dupilumab

IL-4 and IL-13 both have an overlapping and/or distinctive role in T2 immunity related to their receptor expression pattern.

Two forms of IL-4 receptor (IL-4R) are known, the type I and type II IL-4R, which are expressed singularly by the majority of cells in humans, such as B lymphocytes, dendritic cells, monocytes/macrophages, eosinophils, basophils, endothelial cells, bronchial epithelial cells, fibroblasts, and airway smooth muscle cells. On the contrary, the concomitant expression of both forms of the receptor is present in a few cells. The type I receptor is composed of the subunits γ-chain and IL-4Rα, the latter also being part of the structure of the type II receptor, together with the subunit IL-13Rα-1. The first step in the formation of type I IL-4R starts when IL-4 binds with high affinity the subunit IL-4Rα. Then, the complex IL-4/IL-4Rα is recognized by the γ-chain so that, after the hetero-dimerization, the IL-4 signaling is activated. The type II IL-4R can be bound by both the cytokines IL-4 and IL-13 because of the shared subunit IL-4Rα, expressed on hematopoietic and non-hematopoietic cells. In this receptor, the interaction between IL-4Rα and IL-13Rα-1 subunits is allowed after the formation of either IL-4/IL-4Rα or IL-13/IL-13Rα-1 and the hetero-dimerization is necessary to trigger the signaling [25,26,27,28]. The transduction of the signal of both IL-4R involves kinases belonging to the Janus kinase (JAK) family. The cytoplasmic tail of the type I receptor interacts with JAK1 and JAK3, which are activated via auto- and trans-phosphorylation following IL-4/IL-4Rα-induced receptor dimerization and that in turn phosphorylates the substrate molecules signal transducer and activator of transcription 6 (STAT6), insulin receptor substrate 1 (IRS1, hematopoietically expressed), or IRS2 (non-hematopoietically expressed and involved in the development of M2 macrophages). The homodimerization of STAT6, subsequent to its phosphorylation, brings the transcription factor to the nucleus, where it regulates the expression of several genes through the upregulation of GATA3 transcription factor. IRS-2 is a cytoplasmic connector protein that, after phosphorylation, can bind phosphoinositide-3 kinase (PI3K) and growth factor receptor-bound protein 2 (Grb2), which are both involved in the PI3K/AKT signaling pathway mediating Th2 cell population expansion and differentiation of macrophages toward the M2 phenotype [29].

The kinases responsible for type II receptor signaling are JAK1, JAK2, and the tyrosine kinase 2 (TYK2), which can activate STAT6 but not IRS2. Through the activation of STAT6, the receptors mediate the pleiotropic effects of IL-4 and IL-13 in the airways. Recently, experiments on transgenic mice showed that IL-13Rα-1 is fundamental for the development of airway hyperreactivity and mucus hypersecretion, with a milder effect concerning the differentiation of M2 macrophages in the context of asthma [29].

Subsequently, researchers studied different approaches that separately induced the inhibition of IL-4 or IL-13 [30]. In vivo studies in murine models of asthma revealed that both anti-IL-4 antibodies and soluble IL-4 receptors can block the downstream events associated with asthma. Pascolizumab (a humanized monoclonal antibody anti-IL-4) inhibits the interaction between IL-4 and its receptor, provoking the repression of Th2 cell differentiation, eosinophilia, and IgE up-regulation of all processes of the early phase of asthma. These effects were evaluated in a preclinical study that confirmed the potential benefit of pascolizumab as a possible treatment for asthma [31]. Altrakincept (a nebulized, soluble recombinant human IL-4 receptor) was tested on 25 patients with moderate atopic asthma requiring inhaled corticosteroids, but despite the improvement in both lung function and asthma control and the decrease in F_E_NO levels, this drug was not employed in a subsequent phase 3 study [32].

For what concerns asthma therapy through the inhibition of IL-13, two humanized monoclonal antibodies are currently considered [33]. Lebrikizumab is an IgG4 humanized monoclonal antibody that binds to soluble IL-13, preventing the link with its receptor [30]. Different clinical trials were performed to evaluate the efficacy of this biological drug on asthmatic patients. Corren and coworkers conducted a randomized, double-blind, placebo-controlled study in a population of 219 uncontrolled asthmatic patients. They showed that compared with the placebo, patients treated with lebrikizumab (250 mg monthly for 6 months) had a higher increase in FEV_1_. Furthermore, the authors observed that after lebrikizumab treatment, patients with higher levels of serum periostin before treatment had a greater improvement in lung function and reduction of F_E_NO concentrations compared with asthmatics characterized by lower periostin levels [34]. Later, two replicate randomized, double-blind, placebo-controlled phase 2 trials (LUTE and VERSE) confirmed the efficacy of lebrikizumab on uncontrolled asthmatic patients on medium-to-high dose ICS and another controller [35]. The analyses carried out revealed that asthmatics with high periostin had better FEV_1_ levels and a reduction in asthma exacerbations by 60% compared with 5% for patients with low periostin. Unfortunately, the findings observed in asthmatics characterized by higher periostin levels were not replicated in a phase 3 study [36]. The human interleukin-13-neutralizing monoclonal IgG4 antibody called tralokinumab was tested for different diseases, including severe asthma. Pieper et al. conducted a phase 2a, randomized, double-blind, placebo-controlled, parallel-group, multicenter study on 194 uncontrolled moderate-to-severe asthmatic patients [37]. Patients were randomized to receive tralokinumab (150, 300, or 600 mg) or placebo every two weeks. Treatment with tralokinumab as add-on therapy showed no change in the ACQ-6 score but provoked a significant FEV_1_ percentage increase with all doses evaluated, suggesting the clinical efficacy of this biologic in asthmatic populations.

Another randomized, double-blind, placebo-controlled, parallel-group, multicenter, phase 2b study on severe asthmatic patients with two to six exacerbations in the previous year was conducted [38]. The results obtained showed a significant improvement in lung function but not in exacerbation rate reduction; the data were confirmed in the subsequent phase 3 clinical trials [39].

**Figure 2:**
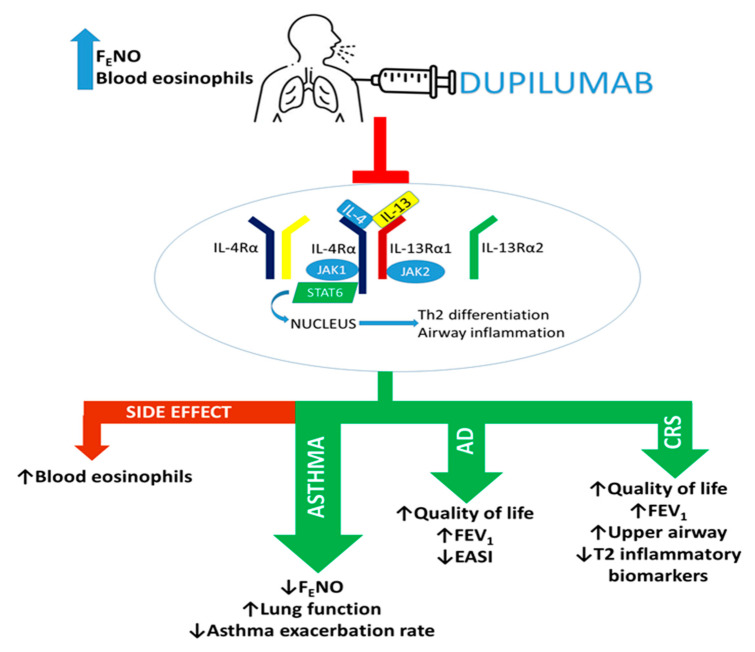
Dupilumab exerts dual blockade of IL-4/IL-13 signaling by binding IL-4Rα. Through this mechanism of action, dupilumab can induce beneficial effects in patients with T2 pathologies. AD: atopic dermatitis, CRS: chronic rhinosinusitis, EASI: Eczema Area and Severity Index, FEV_1_: Forced expiratory volume in the first second, ↓: reduction, ↑: improvement.

Researchers developing a new approach of dual IL-13/IL-4 inhibition (dupilumab), reported a therapeutic achievement with a higher clinical relevance for asthma treatment. Dupilumab binds the IL-4Rα subunit, thus blocking the effects of both IL-4 and IL-13 in the airways (Figure 2) [29].

As stated above, IL-4 and IL-13, through the activation of their receptor, can induce, respectively, the expression of eosinophil chemoattractants (IL-5 and eotaxin-3) and the induction of iNOS, with a subsequent increase in F_E_NO levels.

These mechanisms underlie the observations that dupilumab turned out effective in asthmatic patients with high levels of blood eosinophils and F_E_NO. In more detail, patients with severe asthma receiving high ICS doses are considered eligible for dupilumab treatment when they are characterized by systemic eosinophil counts ≥ 150 cells/µL and F_E_NO ≥ 25 ppb [40].

## 4. Dupilumab Efficacy in Severe Asthma

Recently, a fully human IL-4R-α subunit monoclonal antibody inhibiting IL-4 and IL-13 signaling pathways, called dupilumab, was developed and approved as a treatment of diseases mediated by Th2 pathways. Dupilumab is also approved for the treatment of adults with moderate-to-severe atopic dermatitis and other T2 inflammatory diseases, including nasal polyposis with chronic rhinosinusitis and severe asthma.

The first study that verified the possible therapeutic role of dupilumab in asthma was the work of Wenzel et al., in which the efficacy of dupilumab was studied in adults with persistent, moderate-to-severe asthma that used ICS and long-acting beta-agonists (LABAs) and were characterized by elevated blood and sputum eosinophil levels (≥300 cells/µL and ≥3%, respectively) [41]. This randomized, double-blind, placebo-controlled, parallel-group phase 2a study was conducted on 104 asthmatics treated with dupilumab (300 mg) or placebo for 12 weeks. After dupilumab treatment, the asthmatic patients showed reduced asthma exacerbations rate and β-agonist use, together with improved FEV_1_, ACQ5 score, and asthma symptoms. Furthermore, dupilumab treatment decreased levels of T2 biomarkers, such as F_E_NO, serum IgE, plasma eotaxin-3, and serum thymus and activation-regulated chemokine (TARC), but no changes were observed in blood eosinophils levels. In a later randomized, double-blind, placebo-controlled, parallel-group, pivotal phase 2b clinical trial, Wenzel and colleagues evaluated the efficacy and safety of dupilumab as add-on therapy [42]. This study was conducted on 769 patients with uncontrolled persistent asthma on medium-to-high-dose inhaled corticosteroids plus a LABA. Asthmatics were randomized to receive the following subcutaneous treatment: dupilumab 200 mg every 2 or 4 weeks, 300 mg every 2 or 4 weeks, or placebo. Concerning the entire population, a better therapeutic effect was observed for the 200 and 300 mg treatment at 2 weeks. The analysis also determined the usefulness of dupilumab in patients with elevated concentrations of blood eosinophils (≥300 cells/µL); in fact, the authors identified a major efficacy of dupilumab in terms of improvement of FEV_1_ in patients who showed elevated blood eosinophils and higher ICS dose therapy. Another randomized, double-blind, placebo-controlled, parallel-group trial (QUEST phase 2 study) was performed on 1900 patients with uncontrolled asthma [43]. Patients randomly received add-on dupilumab (200 and 300 mg) or placebo every 2 weeks for 52 weeks. After treatment, asthmatic patients had a significant reduction of asthma exacerbation frequency (50% approximatively), and an improvement, after 2 weeks treatment, of FEV_1_. The efficacy of treatment was highest in patients characterized by elevated levels of systemic eosinophils and F_E_NO (≥150 cells/µL and ≥25ppb, respectively) and resulted in preventing asthma exacerbations and improving FEV_1_. In contrast, no significant clinical effects were observed in patients with lower blood eosinophils and F_E_NO concentrations. Based on these encouraging findings, Busse and co-workers performed a phase 3 international, multicenter, randomized, double-blind, placebo-controlled, parallel-group trial in 1902 patients characterized by uncontrolled moderate-to-severe asthma who were receiving continuous treatment with ICS plus one or two other asthma controller medications [44]. Patients were randomized to receive dupilumab (200 or 300 mg) or placebo every two weeks for 52 weeks. The analysis confirmed the improvement of the clinical outcomes obtained in the previous phase 2 QUEST study, represented by lung function, quality of life, asthma control, and severe exacerbation rate [43].

Rabe and co-workers evaluated whether dupilumab treatment reduces the use of oral glucocorticosteroids (OCS) [45]. This international, randomized, double-blind, placebo-controlled, phase 3 trial (VENTURE study) was conducted on 210 severe asthmatic patients who used OCS to maintain control of the disease. These patients were randomized to receive add-on therapy dupilumab (300 mg) or placebo every 2 weeks for 24 weeks. The analysis revealed a reduction of OCS use without loss of asthma control, a reduction in asthma exacerbation rate, and improvement in lung function in patients treated with dupilumab compared with the placebo group. In the asthmatics who showed higher blood eosinophils (≥300 cells/µL), the treatment with dupilumab led to a 71% lower rate of exacerbations. The findings of the above clinical trials were supported by in vitro and in vivo (murine model of lung inflammation) studies highlighting that the inhibition of IL-4 and IL-13 through dupilumab influenced the activity and expression of effector cells and cytokines that are involved in the propagation of T2 immune response [20].

In a recent study, Corren and colleagues studied dupilumab as a treatment in a cohort of uncontrolled moderate-to-severe allergic asthmatics [46]. The enrolled patients were stratified into two groups: allergic asthma (*n* = 1083) and non-allergic asthma (*n* = 819). These groups were randomized to receive dupilumab (200 or 300 mg) or placebo every two weeks. In the allergic subgroup, dupilumab reduced the annualized rate of severe asthma exacerbations, serum IgE concentrations, and F_E_NO levels and improved ACQ5 score and FEV_1_. The authors found a higher percentage of reduction of an annualized rate of severe exacerbations in asthmatics characterized by levels of IgE ≥ 700UI/mL. This study revealed similar outcomes after dupilumab treatment in non allergic moderate-to-severe asthmatics [46].

Furthermore, Bourdin et al. performed a post hoc analysis on data collected from patients randomized to dupilumab (200 or 300 mg) every 2 weeks and placebo who were enrolled in a phase 2b study (NCT01854047) and the QUEST study [40]. The authors showed that dupilumab (200 or 300 mg) reduced severe asthma exacerbation rate and improved asthma control and FEV_1_ in patients with uncontrolled T2-high persistent or moderate-to-severe asthma taking high-dose ICS at baseline. In addition, this study confirmed that the patients with a high baseline concentration of at least one T2 biomarker, such as F_E_NO (≥25 ppb) and blood eosinophil counts (≥150 or ≥300 cells/µL), had a better outcome in lung function.

Finally, two real-life studies were conducted: the first assessed the effectiveness of dupilumab treatment on a cohort of adult severe asthmatics [47], the second evaluated the benefit of switching to dupilumab those asthmatics who did not achieve control with omalizumab (anti-IgE), mepolizumab or benralizumab [48].

Dupin and coworkers performed a retrospective multicenter study on 64 uncontrolled severe asthma patients and evaluated at 3/6/12 months the improvement of ACT score, FEV_1_, and OCS use. This study was confirmatory of the randomized controlled trials cited above. After treatment at 3, 6, and 12 months, severe asthmatic patients had an increase in asthma control and FEV_1_ and a reduction of OCS dose intake [47].

Another interesting real-life study studied the response of dupilumab in 38 severe asthmatics treated previously with other biologic therapies without achieving a better clinical response. The author demonstrated that 32 severe asthmatic patients had improvement when switched from omalizubam, benralizumab, or mepolizumab to dupilumab. These patients, after 3 to 6 months of treatment, had ameliorated asthma control, improved lung function, and decreased exacerbation rate and F_E_NO and IgE levels [48].

The findings described above confirm, complement, and extend the findings of randomized controlled trials and are useful to better understand how efficacy data could translate to daily clinical practice (Table 1).

## 5. Dupilumab Efficacy in Asthma Comorbidity and Coexisting Conditions

It has been reported that in some patients asthma can co-exist with chronic rhinosinusitis (CRS) or atopic dermatitis (AD) [49,50]. Moreover, patients characterized by uncontrolled and persistent asthma had allergic rhinitis as an atopic comorbidity [51]. A post hoc analysis of the QUEST phase 3 study [43] assessed the effect of dupilumab on 814 uncontrolled moderate-to-severe asthmatic patients with perennial allergic rhinitis, determined by a history of allergic rhinitis and sensitization to one or more perennial aeroallergen-specific IgEs (≥0.35 kU/L) at baseline [52]. The authors reported a rapid improvement in the key standard asthma outcomes analyzed, except for the severe exacerbation rate, for which the effect of dupilumab treatment was lower than for the overall QUEST population. After dupilumab treatment with both doses, patients had a better score with regard to the rhinoconjunctivitis-specific health-related quality of life (HRQoL) questionnaire. Furthermore, according to the dupilumab mechanisms of action, these patients showed a reduction in T2 inflammatory markers, including total serum IgE, F_E_NO, and TARC. These findings confirmed that dupilumab could be helpful in the management of patients with both conditions.

When asthma and CRS are comorbidities, patients present a higher degree of severity and uncontrolled symptoms. CRS phenotypes are divided into disease with and without nasal polyps (CRSwNP and CRSsNP, respectively), with the former characterized by an immunological phenotype associated with a predominant T2 inflammatory response [49,53].

The data derived from the SARP project were elaborated by Denlinger et al., who showed an association between CRS and frequent exacerbation phenotypes of asthma [54]. In several trials, treatment with dupilumab induced a reduction of severe exacerbation rate in asthmatics with or without comorbidities. On this basis, Maspero and co-workers evaluated the efficacy of dupilumab in asthmatic patients with or without self-reported CRS (both CRSwNP and CRSsNP) [55]. The analyses also considered the possible different responses to treatment related to the baseline concentrations of blood eosinophils (≥150 or 300 cells/µL) and F_E_NO levels (≥25ppb). The authors reported that, after treatment, patients with CRS (*n* = 382) showed a more significant reduction in severe asthma exacerbations rate and a higher increase in FEV_1_ than those in the non-CRS group (*n* = 1515). Concerning the effect of dupilumab on the other clinical outcomes, symptoms related to CRS, and T2 biomarkers, the improvements observed were similar between CRS and non-CRS.

Previously, Bachert and colleagues assessed the effects of dupilumab administered as add-on therapy to mometasone furoate nasal spray (MFNS), in patients with CRSwNP and comorbid asthma [56]. The findings of the study revealed that dupilumab improved nasal polyp burden, asthma control, FEV_1_, HRQoL, and patient perception of general health and physical functioning. Similar outcomes were reported in a recent analysis that evaluated the effect of dupilumab on lung function and HRQoL in the patients presenting CRSwNP and comorbid asthma enrolled in the phase 3 SINUS-24 and SINUS-52 trials [57]. In both studies, dupilumab was administered as add-on therapy to MFNS. The authors observed ameliorated FEV_1_ and ACQ-6 score, reduced upper airway obstruction, and improved HRQoL in patients treated with dupilumab compared with patients in the placebo group.

The pathogenic mechanisms of AD are similar to asthma, as they are both Th2-driven diseases with the enhancement of IL-4, IL-5, and IL-13 cytokines that can induce eosinophils and IgE recruitment [50,58].

Available evidence showed the efficacy of dupilumab as monotherapy in patients with moderate-to-severe AD and comorbid asthma, as reflected by a significant reduction in Eczema Area and Severity Index (EASI), a better quality of life, and raised FEV_1_ (Figure 2 and Table 2) [59,60].

## 6. Dupilumab Safety in T2 Diseases

Overall, the clinical trial outcomes revealed that dupilumab is a well-tolerated treatment.

However, all the above-described beneficial effects were accompanied by adverse events (AEs) (Table 3).

Generally, the incidence of the adverse events related to dupilumab treatment was similar across the patient groups assessed in clinical trials.

Injection-site reaction and headache were the most commonly reported AEs during the treatment period, with a higher incidence in patients receiving dupilumab than placebo. Other frequent AEs were upper respiratory tract infections and nasopharyngitis [41,42,43,45,47,55].

A subset of patients under dupilumab treatment had an increase in eosinophils counts, with a greater percentage of asthmatics who achieved eosinophils levels of more than 3000 cells/µL. These transient elevations in the systemic eosinophil counts expired at the end of the dupilumab treatment period (range: 6–12 months) and did not associate with concomitant clinical adverse events or consequences on treatment response or discontinuation of the therapy. The observed rise in blood eosinophilia was hypothesized to be due to the inhibition of IL-4/IL-13 signaling. Dupilumab, by preventing the two cytokines-receptor interactions, hinders the subsequent effects promoting eosinophil proliferation and recruitment [43,45,47]. Therefore, eosinophils can move from the bone marrow to the blood, as this process is mediated by IL-5, but cannot reach the lungs, presumably because of, for instance, a diminished expression of adhesion molecules.

## 7. Dupilumab Effects in Selected Populations

The United States and the European Union approved the use of dupilumab as an add-on therapy for adults and adolescents with T2-driven diseases [61]. Despite the fact that severe asthma in childhood affects a small percentage of patients, children with this disease have a high burden of asthma symptoms. The findings from SARP revealed that children with severe asthma underwent intubation for near-fatal respiratory failure, thus indicating the extent to which severe asthma can be a life-threatening condition for this population [62,63]. The T2 inflammatory response contributes to the pathophysiology of severe pediatric asthma, suggesting a possible efficacy of dupilumab treatment. Several clinical studies are ongoing to assess the efficacy and safety of dupilumab in children with severe asthma and atopic dermatitis [64]. Recently, Paller et al. published the results of the LIBERTY AD PEDS study that was conducted on children (6–11 years) with atopic dermatitis. Dupilumab induced significant improvements in AD symptoms and quality of life and was well tolerated, with a low incidence of adverse events [61]. The outcomes of trials regarding severe asthma have not been reported yet.

There are few data concerning dupilumab treatment during pregnancy because pregnancy represents an exclusion criterion for clinical trials [65]. Preclinical studies revealed no adverse events in maternal animals up to six months post-partum [65]. In 2017, the European Medicines Agency published data from clinical trials that included pregnant female patients who were atopic or were asthmatics treated with dupilumab [66]. The majority of pregnancies resulted in deliveries of healthy babies, but also induced and spontaneous abortions were reported. The rate of spontaneous abortions was not higher than the general spontaneous abortions rate. Further evidence was provided by Kage and co-workers, reporting a case of a woman with atopic eczema treated with dupilumab during pregnancy. The treatment did not provoke teratogenic effects [66].

Elderly patients with atopic dermatitis, severe asthma, and CRSwNP enrolled in clinical trials assessing dupilumab efficacy and safety showed a response to treatment that was similar to that in the younger patients [67].

## 8. Conclusions

Over the last decades, asthma phenotyping allowed the achievement of a deeper comprehension of the molecular mechanisms that are the basis of the plethora of asthma phenotypes. The effort of the researchers also resulted in the development of biological drugs targeting specific pathways that are related to T2 immune response, such as anti-IgE, anti-IL-5, and anti-IL5 receptors. Among them, dupilumab, a fully humanized IgG4 monoclonal antibody, targets IL-4/IL-13 signaling and displays strong efficacy as add-on therapy in severe uncontrolled asthmatic patients as well as in the T2 diseases atopic dermatitis and chronic rhinosinusitis with nasal polyps. Furthermore, it is possible to speculate that the blockage of IL-13 signaling could render dupilumab a drug with beneficial effects on remodeling events that impact the airways of asthmatic patients. This hypothesis is supported by IL-13 actions in the lung, such as goblet cell hyperplasia, smooth muscle cells proliferation, collagen deposition, and fibroblast transformation into myofibroblasts.

Future studies should evaluate the role of dupilumab as a disease-modifying treatment, expanding its usage to a moderate-to-severe asthma population. Should there be a positive response from these future studies, we may imagine a new horizon in asthma, where dupilumab could be considered the first biologic as a first-line drug in selected patients with T2-high asthma and not only as an add-on drug.

Finally, we cannot rule out further exploration of dupilumab in the context of other T2-driven diseases.

## Figures and Tables

**Table 1:** Efficacy of dupilumab in asthma.

	Authors	Population	Summary of Outcomes
DUPILUMAB	Wenzel et al. [41]	104 adults with persistent, moderate-to-severe asthma who used ICS and LABAs, characterized by elevated blood (≥300 cells/µL) and sputum eosinophil levels (≥3%).	↓ asthma exacerbations rate and β-agonist use, improved FEV_1_, ACQ5 score, and asthma symptoms. ↓ levels of F_E_NO, serum IgE, plasma eotaxin-3, and TARC.
	Wenzel et al. [42]	769 patients with uncontrolled persistent asthma on medium-to-high-dose ICS plus a LABA	Improvement of FEV_1_ in patients with blood eosinophils (≥ 300 cells/µL) and higher ICS dose therapy.
	Castro et al. [43]	1900 patients with uncontrolled asthma	Highest efficacy in patients with elevated blood eosinophils (≥150 cells/µL) and F_E_NO (≥25 ppb): preventing asthma exacerbations and improving FEV_1_.
	Busse et al. [44]	1902 patients characterized by uncontrolled, moderate-to-severe asthma who were receiving continuous treatment with ICS plus one or two other asthma controller medications	Improvement in lung function, quality of life, asthma control, and severe exacerbation rate.
	Rabe et al. [45]	210 severe asthmatic patients who used OCS to maintain control of asthma	↓ OCS use without loss of asthma control, ↓ asthma exacerbation rate, and lung function improvement. Asthmatics with higher blood eosinophils (≥300 cells/µL) had ↓ of exacerbations (71%)
	Corren et al. [46]	1902 uncontrolled moderate-to-severe asthmatics: 1083, allergic asthma and 819 non-allergic asthma.	↓ annualized rate of severe asthma exacerbations, serum IgE concentrations, and F_E_NO levels and improved ACQ5 score and FEV_1_ in allergic asthmatic group with IgE levels IgE ≥700 UI/mL. In allergic moderate-to-severe asthmatics, dupilumab determined better clinical outcomes and decreased levels of specific T2 inflammatory biomarkers.
	Bourdin et al. [40]	465 asthmatics who used high (>1000 μg/day)- or medium (500–1000 μg/day)-dose ICS plus LABA; and patients of QUEST study [43]	↓ severe asthma exacerbation rate and improved asthma control and FEV_1_. Patients with a high baseline concentration of at least one T2 biomarker, such as F_E_NO (≥25ppb) and blood eosinophil counts (≥150 or ≥300 cells/µL), had a better outcome in lung function.
	Dupin et al. [47]	64 uncontrolled severe asthma patients	↑ asthma control, FEV_1_, and a reduction of the OCS dose intake.
	Mümmler et al. [48]	38 severe asthmatics treated previously with other biologic therapies without achieving a better clinical response	Asthmatics had improvement when switched from omalizubam, benralizumab, or mepolizumab to dupilumab. After 3 to 6 months of treatment: ↑ asthma control, lung function, and ↓ exacerbation rate and F_E_NO and IgE levels.

ICS: inhaled corticosteroids; LABA: long-acting beta-agonists; OCS: oral corticosteroids; TARC: serum thymus and activation-regulated chemokine; ↓: reduction; ↑: improvement.

**Table 2:** Efficacy of Dupilumab in Atopic Dermatitis and Chronic Rhinosinusitis.

Authors	Population	Summary of Outcomes
Busse et al. [52]	814 uncontrolled, moderate-to-severe asthmatics with perennial allergic rhinitis determined by a history of allergic rhinitis and sensitization to one or more perennial aeroallergen-specific IgEs (≥0.35 kU/L) at baseline	Rapid improvement in the key standard asthma outcomes analyzed, except for the severe exacerbation rate; better score concerning the HRQoL questionnaire; reduction in total serum IgE, F_E_NO, and TARC.
Maspero et al. [55]	1897 asthmatic patients with or without self-reported CRS (both CRSwNP and CRSsNP)	Patients with CRS showed ↓ in severe asthma exacerbations rate and ↑FEV_1_.
Bachert et al. [56]	60 patients with CRSwNP and comorbid asthma	↑ nasal polyp burden, asthma control, FEV_1_, HRQoL, and the patient perception of general health and physical functioning.
Laidlaw et al. [57]	724 patients with CRSwNP with or without comorbid asthma	↑ FEV_1_, ACQ-6 score, HRQoL; ↓ upper airway obstruction,
Benzecry et al. [59]	1 adult patient affected by severe uncontrolled asthma and atopic dermatitis	↑ in Eczema Area and Severity Index, a better quality of life, and raised in FEV_1_
Tolino et al. [60]	1 adult patient affected by severe uncontrolled asthma and atopic dermatitis	↑ in Eczema Area and Severity Index, a better quality of life, and raised in FEV_1_

CRS: chronic rhinosinusitis; CRSwNP: chronic rhinosinusitis with nasal polyps; CRSsNP: chronic rhinosinusitis without nasal polyps; HRQoL: rhinoconjunctivitis-specific health-related quality of life; TARC: serum thymus and activation-regulated chemokine; ↓: reduction; ↑: improvement.

**Table 3:** Adverse events of dupilumab treatment.

Dupilumab Dosage	Adverse Events
300 mg once weekly [41]	Injection-site reaction
200 or 300 mg every 2 or 4 weeks [42,55]	Headache
200 or 300 mg every 2 weeks [43]	Upper respiratory tract infections
300 mg every 2 weeks [45,47]	Nasopharyngitis
200 or 300 mg every 2 weeks [43]	Increase in eosinophils counts
300 mg every 2 weeks [45,47]	
300 mg every 2 weeks [45,47]	

## Data Availability

Not applicable.
